# Sentiment Analysis of Health Care Tweets: Review of the Methods Used

**DOI:** 10.2196/publichealth.5789

**Published:** 2018-04-23

**Authors:** Sunir Gohil, Sabine Vuik, Ara Darzi

**Affiliations:** ^1^ Imperial College London Department of Surgery and Cancer London United Kingdom

**Keywords:** Twitter, social media

## Abstract

**Background:**

Twitter is a microblogging service where users can send and read short 140-character messages called “tweets.” There are several unstructured, free-text tweets relating to health care being shared on Twitter, which is becoming a popular area for health care research. Sentiment is a metric commonly used to investigate the positive or negative opinion within these messages. Exploring the methods used for sentiment analysis in Twitter health care research may allow us to better understand the options available for future research in this growing field.

**Objective:**

The first objective of this study was to understand which tools would be available for sentiment analysis of Twitter health care research, by reviewing existing studies in this area and the methods they used. The second objective was to determine which method would work best in the health care settings, by analyzing how the methods were used to answer specific health care questions, their production, and how their accuracy was analyzed.

**Methods:**

A review of the literature was conducted pertaining to Twitter and health care research, which used a quantitative method of sentiment analysis for the free-text messages (tweets). The study compared the types of tools used in each case and examined methods for tool production, tool training, and analysis of accuracy.

**Results:**

A total of 12 papers studying the quantitative measurement of sentiment in the health care setting were found. More than half of these studies produced tools specifically for their research, 4 used open source tools available freely, and 2 used commercially available software. Moreover, 4 out of the 12 tools were trained using a smaller sample of the study’s final data. The sentiment method was trained against, on an average, 0.45% (2816/627,024) of the total sample data. One of the 12 papers commented on the analysis of accuracy of the tool used.

**Conclusions:**

Multiple methods are used for sentiment analysis of tweets in the health care setting. These range from self-produced basic categorizations to more complex and expensive commercial software. The open source and commercial methods are developed on product reviews and generic social media messages. None of these methods have been extensively tested against a corpus of health care messages to check their accuracy. This study suggests that there is a need for an accurate and tested tool for sentiment analysis of tweets trained using a health care setting–specific corpus of manually annotated tweets first.

## Introduction

Today’s doctors and patients take to online platforms such as blogs, social media, and websites to convey opinions on health matters [1]. Infodemiology is “the science of distribution and determinants of information in an electronic medium, specifically the Internet, or in a population, with the ultimate aim to inform public health and public policy” [2]. Data can be collected and analyzed from social media such as Twitter in real time with the ability to survey public opinion (sentiment) toward a subject [3]. Bates and colleagues have described social media as a “perfect storm” in regard to patient-centered health care, which is a valuable source of data for the public and health organizations [4]. Twitter is one such place, being easy to use, cheap, and accessible. Twitter is a mobile microblogging and social networking service. There are currently 955 million registered Twitter users who can share messages that contain text, video, photos, or links to external sources. One-third of people with a social media profile use Twitter, with 75% accessing from a handheld device to convey an opinion [5,6].

Sentiment analysis allows the content of free-text natural language—that is, the words and symbols used in a message—to be examined for the intensity of positive and negative opinions and emotions. Sentiment analysis from social media is already a widely researched subject [7]. It is useful for business marketing to understand the public or consumer opinion toward their product [8]. Computerized software tools have been produced that automate the process of sentiment analysis, allowing large numbers of free-text comments to be processed into quantitative sentiment scores quickly, for example, positive or negative [7]. They are commonly based on text classifiers or machine learning processes. These tend to be commercially orientated, expensive, and focused on gathering opinion on a specific chosen product or service [9]. During the H1N1 outbreak, Chew et al conducted a content analysis of tweets [10]. In this study, they measured sentiment in a qualitative categorical way using content classifiers such as “humor” or “sarcasm.” Accurate and automated sentiment analysis is challenging due to the subjectivity, complexity, and creativity of the language used [11].

Sentiment analysis in the health care setting is not a new phenomenon. Using only manual annotation of health care tweets, it has been found that 40% of messages contain some form of sentiment (either positive or negative) [12]. A manual method has also been used in the analysis of suicide notes and discharge summaries, where Cherry et al attempt to automate the manual process using machine learning approaches [13-15]. It was found that the manual classification of emotional text was difficult and inconsistent [13]. Greater positive sentiment within discharge summaries was associated with significantly decreased risk of readmission [14]. A study was also conducted measuring the sentiment of comments on the main National Health Service (NHS) website (NHS choices) over a 2-year period [16,17]. They found a strong agreement between the quantitative online ratings of health care providers and analysis of sentiment using their automated method.

Sentiment analysis has made its way into the mainstream analysis of Twitter-based health care research. Twitter is a popular platform as it allows data to be collected easily using their application programming interface. The limitations of other social media platforms such as Facebook are they do not allow such easy access to their data due to their varying privacy policies. It is not as easy to collect data in an open and automated way with other such media. The opinion of a tweet is found within the text portion of the tweet. This is captured in an unstructured, nonstandardized, free-text form. Accurately measuring the sentiment of a health care tweet represents an opportunity for understanding both the patient’s and health care professional’s opinion on a health subject [16]. Kent et al found that up to 40% of health care tweets contain some form of sentiment [12]. A validated tool for sentiment analysis of health care messages on Twitter would allow for the assessment of opinion on a mass scale [17]. Sentiment analysis in the medical setting offers a unique challenge as terms can have varying usage and meanings, and requires complementary context-specific features with a domain-specific lexicon [18]. The language used to convey sentiment in medicine is likely to be different than that toward a product, as the boundary between “patient,” “consumer,” and “customer” is difficult to define and terms can have varying usage and meanings [11,19]. Therefore, the sentiments may be expressed differently in a health care context [18].

To date, there has been no study looking at all the methods used for sentiment analysis on Twitter in the health care setting. Currently available sentiment analysis tools have not been developed based on a health care setting. SentiStrength [20], a popular open source software was based on nonspecific messages sent via MySpace [21]. Health care can be a very different environment based on many aspects. Being a public National Health Service [19], the boundary between “patient,” “consumer,” and “customer” is difficult to define in health care Therefore, currently available sentiment analysis methods may not be accurate.

The aim of this study was to review the methods used to measure sentiment for Twitter-based health care studies. The first objective was to review what methods of sentiment analysis have been used and in which health care setting. The second objective was to explore to what extent the methods were trained and validated for the study data, and if any justification for their methodology use was offered.

## Methods

### Identification and Screening

In May 2015, a computerized search of the literature was conducted, following Preferred Reporting Items for Systemic Reviews guidelines [22]. MEDLINE (OvidSP) and EMBASE (OvidSP) were searched using the terms. References were checked from papers and reviews, and citations were checked from included studies. The titles and abstracts were screened from the retrieved search to identify relevant studies. A supplementary hand search was carried out in September 2016 in key journals. Studies had to include one of the following search terms in the title, abstract, or keywords: “Twitter” or associated terms “tweet” or “microblog” and “Sentiment” or associated search terms “opinion” or “emoti” or “happi” or “Senti.” There were 3 inclusion criteria for the study. First, the study must have Twitter as its primary focus. The aim of this review was to explore research into the methods of sentiment analysis on Twitter messages only. Second, the papers must be relating to a health care subject. This included all aspects of health and health care delivery, health care research, policy, and organizational and professional use. Finally, papers that used a quantitative method to analyze both positive and negative sentiments of the messages, for example, “−1,” were included.

### Eligibility and Inclusion

The studies were restricted to those published in English. A total of 69 full-text articles were assessed for eligibility. Of these, 15% (10/69) were rejected because they looked at social media in general (not Twitter specifically), for example, the use of social media by surgical colleagues [23]. Moreover, 36% (25/69) were rejected because the study did not pertain to health care, for example, public perceptions of nonmedical use of opioids [24]. Furthermore, 32% (22/69) papers were excluded because the sentiment analysis was either not measured, not quantitative or did not discuss positive and negative sentiments specifically, for example, characterizing sleep issues using Twitter [25]. The criteria used to compare the methods in each study looked at the method of tool production, in which setting it was used, and the method of testing the tool. For assessment, a comparison of the number of annotators used to manually annotate tweets, if any, and the level of agreement between them was used. Furthermore, the proportion of tweets used to train an algorithm compared with the final sample analyzed was also assessed.

## Results

### Overall Results

In total, 12 papers were found that satisfied all 3 inclusion criteria (see Table 1 for overview). These were published between 2011 and 2016 with data collected from Twitter between 2006 and 2016. Moreover, 2 papers examined global data, 9 in the United States, and 1 in the United Kingdom. Comments from 2 papers suggest that on an average 46% (92/2) of health care tweets contain some form of sentiment, that is, not neutral [12,26]. Many studies conducted analysis on public health–related subjects (n=7). In addition, 3 papers examined the sentiment toward an aspect of disease: the disease itself (n=1), symptoms (n=1), or treatment (n=1). Finally, 2 papers studied an emergency medical situation and a medical conference.

A total of 5 of the 12 studies conducted a manual sentiment analysis of a sample of their data using annotators to train their tool. One study used 13.58% (1000/7362) of their final data sample to train their developed method [34]. Three studies used an average of 0.7% of their total dataset to train their tool (1.46%, 250/17,098; 0.55%, 2216/404,065; and 0.1%, 250/198,499). One paper compared the accuracy of their chosen methods with a manually annotated corpus of their data [30]. Moreover, 2 papers from the group commented on justification of the sentiment analysis tools used.

There were 3 categories of sentiment analysis methods found (see Table 2), a tool specifically produced and trained for that study data, open source tools, and commercially available software. This distinction was made based on the required level of expertise in computer programming needed to implement that method and if predefined lexicons were used. Tools produced specifically for the study required the most amount of programming knowledge as these sometimes required the use of machine learning techniques to train a tool or rule-based methods. Alternatively, using commercially available software required the least knowledge as these are designed to be quick and easy to use. Half of the studies conducted quantitative sentiment analysis using an automated method developed by the study group themselves using algorithms or machine learning techniques. Moreover, 3 studies used commercially available sentiment analysis products. The remaining 3 papers used open source, freely available sentiment analysis software, which required little programming experience. In addition, 1 study from the open source and 1 from commercial method studies used a method of manual training to tailor the tool for their specific study data [33].

**Table 1:** Tools used for sentiment analysis.

Author	Year	Location	Subject area	Sentiment toward	Type of method
Bhattacharya et al [27]	2012	United States	Public health	25 Federal health agencies	Open source
Black et al [28]	2011	United States	Emergency medicine	2011 Japanese earthquake and tsunami	Commercial
Cole-Lewis et al [29]	2013-2014	United States	Public health	Electronic cigarette	Produced for study
Daniulaityte et al [30]	2016	United States	Public health	Sentiment toward drug-related tweets	Produced for study
Desai et al [31]	2011	United States	Medical conference	Twitter activity at Kidney Week 2011	Produced for study
Greaves et al [32]	2012	United Kingdom	Public health	Hospital quality	Commercial
Hawkins et al [33]	2015	United States	Public health	Hospital quality	Open source
Myslin et al [34]	2012	Global	Public health	Tobacco	Produced for study
Nwosu et al [35]	2015	Global	Disease specific	Palliative medicine	Open source
Ramagopalan et al [26]	2006-2014	United States	Disease treatment	Multiple sclerosis treatments	Open source
Sofean and Smith [36]	2013	United States	Public health	Tobacco	Produced for study
Tighe et al [37]	2015	United States	Disease symptoms	Pain	Produced for study

**Table 2:** Sentiment tools based on type of tool: KNN: k-nearest-neighbors; N/A: not applicable; NB: Naïve Bayes; SVM; support vector machines.

Author	Tool	Annotators	Kappa	Manually annotated sample	Sample size	Manually annotated compared with total sample, n (%)
Cole-Lewis et al [29]	Produced for study: machine learning classifiers based on 5 categories (NB, KNN, and SVM)	6	.64	250	17,098	250 (1.46)
Desai et al [31]	Produced for study: rule based using AFINN (Named after the author, Finn Arup Neilsen)	N/A	N/A	N/A	993	N/A
Daniulaityte et al [30]	Produced for study: logistic regression, NB, SVM	2	.68	3000	N/A	N/A
Myslin et al [34]	Produced for study: machine learning (NB, KNN, SVM)	2	>.7	1000	7362	1000 (13.58)
Sofean and Smith [36]	Produced for study: 5-fold validation using support vector machines (SVM’s) model using Waikato Environment for Knowledge Analysis toolkit toolkit	N/A	N/A	500	N/A	N/A
Tighe et al [37]	Produced for study: rule based using AFINN	N/A	N/A	N/A	65,000	N/A
Bhattacharya et al [27]	Open source: SentiStrength	3	N/A	N/A	164,104	N/A
Hawkins et al [33]	Open source: machine learning classifier using Python library TextBlob	2+Amazon Mechanical Turk	>.79	2216	404,065	2216 (0.55)
Ramagopalan et al [26]	Open source: TwitteR R package + Jeffrey Breen’s sentiment analysis code	N/A	N/A	N/A	60,037	N/A
Black et al [28]	Commercial: radian6	N/A	N/A	N/A	N/A	N/A
Greaves et al [32]	Commercial: TheySay	N/A	N/A	250	198,499	250 (0.13)
Nwosu et al [35]	Open source: TopsyPro	N/A	N/A	N/A	683,500	N/A

A total of 5 studies commented on the number of annotators used for the manual classification of sentiment to train their final tool (average=3 annotators, range 2-6). A single study used a method of outsourcing the task of manual classification to multiple anonymous annotators via Amazon Mechanical Turk [38].

### Self-Produced Sentiment Analysis Tools

Of the 12 studies reviewed, 6 produced sentiment analysis tools within their own department, specifically designed for their study using already defined algorithms. Liu describes the different types of algorithms that can be used, and they produce different kinds of summaries [39,40]. Moreover, 2 different types of algorithms were found to be used, a standard supervised machine learning algorithm and a classification method (such as AFINN named after the author, Finn Arup Neilsen). These methods produce their own classifier trained to detect polarity using their original data. These may be different from the open source tools, which use already pretrained classifiers in premade software systems designed more toward an end user.

A total of 3 papers used a similar method of sentiment via categorization, all examining opinions toward smoking. Sofean et al produced an automated sentiment tool based on identifying 250 positive and 250 negative tweets from a smaller sample to train their tool [36]. There was no further detail into the annotation and analysis process. A limitation to their tool was that it screened out emoticons (symbols used to express emotion) before producing a tool. This is a method often used by users to convey emotion [39]. Myslin et al analyzed the sentiment toward emerging tobacco products on 7362 tweets, where Cole-Lewis et al looked specifically at sentiment toward electronic cigarettes on 17,098 tweets [29,34]. Neither of the studies commented on why a self-produced solution was used. Tweets were broadly categorized into “positive,” “neutral,” or “negative” by the annotators. The intensity of the sentiment was not recorded. To find the relationship between the sentiment and subject, 3 machine learning algorithms were used, Naïve Bayes, K-Nearest-Neighbor, and Support Vector Machine [41]. An automated sentiment analysis tool was produced based on the manual analysis of sentiment of a sample of tweets during the pilot phase of each study. This represented 13.58% (1000/7362) for Myslin. The study by Cole-Lewis used only 1.46% (250/17,098) of their total sample to train their algorithms. This represents a very small percentage of their sample and may result in their method being less accurate than intended. However, no comment is made by the study group to why only this number was used.

Desai et al used the AFINN (named after the author, Finn Arup Neilsen), to measure the sentiment of Twitter activity during Kidney Week 2011 from 993 tweets [31]. AFINN is a rule-based approach combined with statistical modeling to create a hybrid approach to sentiment classification [7]. This is based on comparing a sample of data with a list of weights of positive or negative keywords using the affective norms for English words dataset [42]. The AFINN consists of a list of manually labeled English words that have been given an integer value between −5 (highly negative) to +5 (highly positive). A value is assigned for each word in a tweet using the lexicon. The values are averaged to calculate the sentiment score for the whole message. This method has been validated for use in microblogs such as Twitter [43]. Tighe et al used this method to assess the sentiment of tweets pertaining to pain, suggesting a rule-based classifier has greater methodological advantage due to its deterministic results compared with human annotators which can have poor interannotator agreement with sentiment [37]. In addition, they supplemented AFINN with the use of emoticon terminology to enhance the accuracy of the rule-based classifier [39,44]. One study sought to compare different supervised machine learning (SML) techniques with each other, and to a rule-based open source lexicon for drug-related tweets [30]. They found that by using manually annotated tweets specifically from that subject to train SML techniques was more accurate than a preprepared lexicon due to the variation in language used. They also compare types of SML techniques to show that they all performed to a similar level.

### Open Source Sentiment Software

Open source software is a computer software that has its source code made available to the public to modify [45]. The developers or copyright holders of the software give the rights to study and distribute the software for any purpose for free. Moreover, 4 papers used open source software for their sentiment analysis. None of these tools were initially produced using health care messages. Ramagopalan et al investigated the opinions of specific multiple sclerosis treatments using 60,037 tweets [26]. They used an open source sentiment analysis tool called package twitteR R [46] in combination with Jeffrey Breen’s sentiment analysis code [47]. This software was developed for the analysis of consumer sentiment toward a product and compares the frequency of positive or negative words against a predefined list. The overall sentiment score of each message is calculated by subtracting the number of negative words from the number of positive words. A sentiment score of >0 suggests that the message has an overall positive opinion. Of their dataset, 52% of messages contained a non-neutral sentiment. This study showed that there was a statistically significant difference in sentiment toward different types of multiple sclerosis medications. There was no comment on analysis of the tool itself or justification of its use.

Bhattacharya et al used SentiStrength [20,48], a popular open source software to analyze the sentiment of 164,104 tweets from 25 Federal Health Agencies in the United States and their 130 accounts. SentiStrength has been designed to measure the sentiment of short informal messages and has been widely used for Twitter analysis [49]. It was used in this case because it outperforms other lexical classifiers [42]. No manual sentiment analysis was conducted.

SentiStrength was developed in 2009 to extract sentiment strength from informal English text, giving a rating between −5 and +5. The algorithm was developed on an initial set of 2600 MySpace comments used for pilot testing. A set of 3 same gender (female) coders were used for initial testing and this was optimized by machine learning into its final version. It can detect positive emotion with 60.6% accuracy and negative emotion with 72.8% accuracy. SentiStrength outperforms a wide range of other machine learning approaches. SentiStrength has not yet been validated specifically for health care–based messages.

Hawkins et al measured patient-perceived quality of care in US hospitals using Twitter [33]. Over 404,000 tweets were analyzed for their sentiment and compared with established quality measures over a 1-year period. Natural language processing was used to measure the sentiment of the patient experience tweets. This was based on a Python library TextBlob [50]. TextBlob is trained from human annotated words commonly found in product reviews based on the Pattern Library [51]. The sentiment score can range from −1 to +1, with a score of 0 suggesting a tweet that is neutral. This was the first study that adopted Amazon Mechanical Turk [38] to use multiple outsourced anonymous curators to train their tool. They found a weak association between the positive sentiment toward a hospital and the readmission rate.

### Commercial Software

There are numerous commercial software packages available to analyze the sentiment of tweets. These range in price depending on the number of tweets or duration of use. In this study, 2 papers were found using commercial software. Neither tool was developed with health care messages as its foundation, and no justification for their use is offered for either.

The largest number of messages analyzed by Nwosu measured the sentiment of over 683,000 tweets based around palliative medicine and end of life care [35]. Discussion about end of life can be difficult and sometimes missed [52]. TopsyPro was used to measure the sentiment of tweets [53]. This software was created in 2015 as an Web based tool for Twitter analytics and sentiment analysis and is based on an annual subscription costing US $12,000 per year per named user (for the “Pro” version which enables more detailed analysis). There is no information currently available on the methods used by Topsy Labs, Inc. on how the sentiment analysis is conducted.

Radian6 [54] is another piece of “listening” social media software to collect and analyze data. It has been previously used to collect data during a medical conference, with analysis focused on the major Twitter influencers [55]. The software does not require the user to have any programming knowledge and is deigned to be easy to use. Black et al used this software to analyze tweets based around public health emergency response during the Japanese earthquake and tsunami in March 2011. There was no comment on why this software was used. Radian6 can “listen” automatically to large-scale Twitter conversation based on specific keywords.

A study conducted by Greaves et al was found looking at hospital quality in the United Kingdom, and it measured the sentiment of over 198,000 tweets directed toward NHS hospitals in 2012 [32]. The commercially available software used was developed by TheySay Ltd (Oxford, UK). TheySay is based on compositional sentiment parsing, described by work from Moilanen and Pulman, using 5 automated ways of natural language processing [56]. For academic purposes, the software costs roughly £350 for a similar volume of data to the mentioned study to be analyzed.

## Discussion

### Principal Findings

On average, 46% (92/2) of health-based tweets contain some form of positive or negative sentiment [12,26]. A relationship between sentiment on Twitter and hospital statistics has already been proven [33]. It is important to conduct sentiment analysis for health care tweets that is accurate and consistent. This study has found that there is a large disparity in the types of methods used, from basic categorizations to seemingly sophisticated and expensive commercially available software. Between the same subject matter such as hospital quality, different sentiment analysis methods have been used which makes it difficult to compare the results between the two [32,33]. Chew et al conducted a content analysis of tweets during the 2009 H1N1 outbreak and chose to use only a qualitative method for sentiment analysis of tweets, categorizing tweets based on emotive words, for example, “Humour” or “Concern” [10]. On the basis of complexity of implementation, 3 broad categories of methods have emerged: (1) self-produced methods using algorithms, (2) open source methods, and (3) commercially available software. Only 1 method in this study was produced with health care language as its foundation using a corpus of manually annotated health care setting–specific tweets for training [30]. Many methods were based on tools trained on product reviews and nonspecific social media messages that may not be appropriate for use in the health care setting [20,57]. The language used to convey sentiment in medicine is likely to be different than that toward a product as the boundary between “patient,” “consumer,” and “customer” is difficult to define and terms can have varying usage and meanings [11,18,19]. Health-related tweets represent a unique type of content, and their communication on Twitter carries special characteristics as found in pain-related tweets [37].

Most studies did not justify the reason for their selected method. Furthermore, there was no evidence of analysis of accuracy of the method before being used for the larger respective data. Researchers tend to assume a method selected will be accurate. Most self-produced methods train their tool using a very small percentage of their final dataset, in one case less than 2% [29]. A formal process for checking the accuracy occurred in one of the author’s study that compared types of supervised machine learning techniques. Software products and open source tools being currently used tend to be designed originally to identify opinions about products in the commercial setting rather than behaviors. This questions their accuracy when used in a medical setting.

### Recommendations

This research shows that different approaches are used for the sentiment analysis of tweets in the health care setting. The evidence suggests that there is a need for the production and analysis of accuracy of a sentiment analysis tool trained using setting-specific health care tweets. Twitter is used globally, and health care can vary greatly depending on the setting. On the basis of this study, such a tool would ideally be trained using a health care subject-specific corpus of labeled tweets to train supervised machine learning classifiers [30]. Semantic Evaluation Exercises (SemEval 2016) held in San Diego is an event where programmers are tasked with producing a sentiment analysis tool on a range of Twitter subjects such as a political candidate or product, using a pre-annotated corpus. This collaborative approach could be used to produce a more advanced and accurate tool for the health care setting using subject-specific lexicons and complementary health care–based features [11,18,58]. Furthermore, it could measure the intensity of sentiment using an aggregation of methods (eg, emoticons, natural language processing, and supervised machine learning), and it could check for accuracy against a slightly larger manually annotated dataset before being used on much larger sample sizes. This could allow future research in health care–based tweets to accurately and consistently measure the sentiment of setting specific health care–based messages.

## Abbreviations

KNN: k-nearest-neighbors
NB: Naïve Bayes
NHS: National Health Service
SML: Supervised Machine Learning
SVM: support vector machines
